# Cardiovascular response to dobutamine stress predicts outcome in severe sepsis and septic shock

**DOI:** 10.1186/cc6814

**Published:** 2008-03-04

**Authors:** Anand Kumar, Elizabeth Schupp, Eugene Bunnell, Amjad Ali, Barry Milcarek, Joseph E Parrillo

**Affiliations:** 1 Section of Critical Care Medicine, Health Sciences Centre/St. Boniface Hospital, University of Manitoba, Winnipeg, Manitoba, Canada; 2 Division of Cardiovascular Disease and Critical Care Medicine, Cooper University Hospital, Robert Wood Johnson Medical School, Camden, New Jersey, USA; 3 Division of Cardiovascular Disease and Critical Care Medicine and Rush-Presbyterian-St. Luke's Medical Center, Chicago, Illinois, USA; 4 Section of Nuclear Medicine, Rush-Presbyterian-St. Luke's Medical Center, Chicago, Illinois, USA

## Abstract

**Introduction:**

During septic shock, resistance to the haemodynamic effects of catecholamine vasopressors and inotropes is a well-recognised marker of mortality risk. However, the specific cardiovascular or metabolic response elements that are most closely associated with outcome have not been well defined. The objective of this study was to assess cardiovascular and metabolic responses to dobutamine as correlates of outcome in patients with severe sepsis or septic shock.

**Methods:**

A prospective, non-randomised, non-blinded interventional study of graded dobutamine challenge (0, 5, 10, and 15 μg/kg/min) in adult patients who had undergone pulmonary artery catheterisation within 48 hours of onset of severe sepsis or septic shock (8 survivors/15 non-survivors) was performed. Radionuclide cineangiography during graded infusion was used to determine biventricular ejection fractions at each increment of dobutamine.

**Results:**

In univariate analysis, a variety of cardiovascular or haemodynamic and oxygen transport or metabolic variables (at the point of maximum cardiac index response for a given subject) were associated with survival including: increased stroke volume index (p = 0.0003); right ventricular end-diastolic volume index (p = 0.0047); left ventricular stroke work index (p = 0.0054); oxygen delivery index (p = 0.0084); cardiac index (p = 0.0093); systolic blood pressure/left ventricular end-systolic volume index ratio (p = 0.0188); left ventricular ejection fraction (p = 0.0160); venous oxygen content (p = 0.0208); mixed venous oxygen saturation (p = 0.0234); pulse pressure (p = 0.0403); decreased pulmonary artery diastolic pressure (p = 0.0133); systemic vascular resistance index (p = 0.0154); extraction ratio (p = 0.0160); and pulmonary vascular resistance index (p = 0.0390). Increases of stroke volume index of greater than or less than 8.5 mL/m^2 ^were concordant with survival or death in 21 of 23 cases. Multivariate profile construction showed stroke volume index as the dominant discriminating variable for survival with the systolic blood pressure/left ventricular end-systolic volume index ratio alone among all other variables significantly improving the model.

**Conclusion:**

Survivors maintain cardiac responsiveness to catecholamine stimulation during septic shock. Survival from severe sepsis or septic shock is associated with increased cardiac performance and contractility indices during dobutamine infusion. Further studies are required to determine whether these parameters are predictive of outcome in a larger severe sepsis/septic shock population.

## Introduction

The cardiovascular profile of septic shock has been well defined over the last 30 years. One of the major areas of investigation has been the cardiovascular correlates of outcome. Early clinical studies, prior to the widespread adoption of the balloon-tipped pulmonary artery flotation catheter with thermodilution capability in the 1980s, tended to suggest that fatal sepsis and septic shock were characterised by a low cardiac output (CO) and high systemic vascular resistance (SVR) [1]. However, the concept of septic shock as a hyperdynamic circulatory state characterised by high cardiac output and low systemic vascular resistance eventually became well established [2,3]. Several baseline cardiovascular variables have been proposed to differentiate between survivors and non-survivors of septic shock including heart rate (HR), CO, SVR and oxygen delivery index (DO_2_I) [4-7]. Studies using radionuclide cineangiography and volumetric echocardiography have further demonstrated an association between survival and biventricular dilatation and ejection fraction (EF) depression [3,8-10]. Evidence has also accumulated suggesting that fatal septic shock is associated with decreased responsiveness to fluid resuscitation [11,12].

Bedside experience and several clinical studies suggest that blunted cardiovascular responsiveness to catecholamine support is also a marker of high risk of death in sepsis and septic shock [13]. However, the relationship of ventricular ejection fraction and/or ventricular volume responses and other ventricular volume-related cardiovascular variables under dobutamine stress to survival in sepsis and septic shock is undefined. This study was designed to assess ventricular and cardiovascular performance, oxygen transport, and oxygen delivery responses during dobutamine challenge and their association with survival in sepsis.

## Materials and methods

This study received Institutional Review Board approval. Informed consent was obtained from all subjects or appropriate representatives prior to enrolment.

Adult (more than 18 years of age) Intensive Care Unit patients were recruited into this prospective, non-randomised, open interventional study. In order to be eligible, patients were required to have developed severe sepsis or septic shock within the preceding 48 hours and to have both a pulmonary artery catheter and arterial pressure catheter in place (based on clinical requirements as defined by the attending physician). Study eligibility was further contingent on confirmation of correct placement of the pulmonary artery catheter based on appropriate pressure traces on insertion and position on chest radiography.

The presence of severe sepsis was defined by 1991 SCCM/ACCP Consensus Statement on Sepsis Definitions [14]. These criteria included: the presence of suspected or documented infection; three of the following four systemic inflammatory response elements temperature >38°C or <36°C, heart rate >90 beats/min, respiratory rate >20 breaths/min or PaCO_2 _<32 mm Hg and white blood cell count >12,000 cells/mm^3^, <4,000 cells/mm^3 ^or >10 per cent immature (band) forms; and evidence of at least one organ failure.

Septic shock was defined by the presence of post-fluid resuscitation hypotension (mean arterial pressure <65 mmHg) necessitating vasopressor support.

Patients with known ischemic cardiac disease and mixed shock states with clinically significant primary cardiogenic, haemorrhagic or pulmonary embolic elements were excluded.

After consent was obtained, each patient underwent graded dobutamine challenge as follows: If required, subjects initially received saline infusion to a pulmonary artery wedge pressure (PWP) of 15 mmHg as required because pilot studies had shown the potential for significant hypotension with dobutamine absent adequate intravascular volume as evidenced by PWP of 15 or higher. After documentation of a PWP of 15 mmHg or higher, baseline haemodynamic and vital parameters (on clinically indicated doses of vasopressors) were obtained. These included heart rate (HR), systemic arterial pressures, central venous pressure (CVP), pulmonary artery pressure (PAP), pulmonary wedge pressure (PWP) and CO. Thermodilution cardiac outputs were measured by the averaging of three of four successive injections of 10 mL of cold (6 to 10°C) dextrose 5 per cent in water at end-expiration (with the outlying values discarded). Recorded values for PAP, PWP and right atrial pressure (RAP) were also obtained at end-expiration from graphic recordings. Right and left ventricular ejection fractions (EFs) were assessed through first-pass gated technetium^99 ^cineangiography as described below. In addition, simultaneous arterial and mixed venous blood samples (distal pulmonary artery port) were obtained for analysis of the serum pH, partial pressure of oxygen (pO_2_), partial pressure of carbon dioxide (pCO_2_), and oxygen saturation (Bayer-CIBA Model 865, Tarrytown, NY).

After baseline values were obtained, a graded dobutamine challenge was performed. Dobutamine infusion through the pulmonary artery catheter introducer port was initiated at 5 μg/kg/min. After 15 minutes, repeat non-invasive and invasive haemodynamic parameters were obtained over approximately five minutes. At this time, subjects also underwent repeat first pass radionuclide assessment of biventricular ejection fractions and arterial and mixed venous blood gas analysis. The dobutamine infusion rate was increased by 5 μg/kg/min at 20 minute intervals to a maximum of 15 μg/kg/min with the assessment process repeated during the final five minutes of each interval. During the infusion, fluid and additional vasopressor support (beyond baseline needs) to maintain a minimum PWP of 15 mmHg and a mean arterial pressure (MAP) of 65 mmHg was mandated; however, no subject required such support. During the period of the study environmental stimulation was minimised. All subjects were continuously monitored with continuous electrocardiographic (ECG) monitoring and pulse oximetry according to standard protocol in the intensive care unit. The protocol required discontinuation of dobutamine if any transient adverse effects were noted including significantly increased ventricular ectopy or decreased blood pressure. After study completion, dobutamine was tapered over 20 minutes and then discontinued. The patient's subsequent intensive care and hospital course were directed by normal clinical practice.

### Radionuclide Cineangiography

Sequential measurement of biventricular ejection fraction was performed by repeat first-pass radionuclide cineangiography using technetium^99^-DPTA. Tc^99^-DPTA was injected as a tight bolus into the central veins using the pulmonary artery catheter introducer. In this study, the baseline radionuclide tracer dose was 2 mCi with subsequent increments to 5, 12 and 25 mCi. The study was performed in a 30° right anterior oblique projection with a slant hole collimator fitted on to a small field gamma camera interfaced with a dedicated computer system (ICON, Siemens, Gammasonic). The data was acquired in frame mode with 440 frames, each of 60 milliseconds duration. The first transit cardiac data was reformatted into a multi-gated study using the subject's electrocardiogram recorded with the first pass data. This method provides independent cinematic display of the right as well as left ventricle. Ejection fractions (EFs) are calculated from the reformatted gated first pass studies using standard dual region of interest and background correction [15,16].

Stroke volume (SV) was derived by dividing thermodilution CO by the concomitant HR. End-diastolic volume (EDV) was obtained by dividing SV by EF and end-systolic volume (ESV) was calculated as EDV-SV. SVR was calculated as 79.9(MAP-RAP)/CO and pulmonary vascular resistance (PVR) as 79.9(mPAP-PWP)/CO where MAP = mean arterial pressure and mPAP = mean pulmonary artery pressure. Where appropriate, cardiovascular variables were indexed to body surface area.

### Data analysis

Haemodynamic and oxygen transport/consumption variables at the baseline and at each dobutamine infusion rate were pooled for all subjects to derive means and standard errors of the mean. The infusion rate at the point of maximum cardiac index (CI) during dobutamine administration was determined for each subject and the corresponding haemodynamic/clinical/oxygen metabolism values at that point were used for comparison to baseline (before dobutamine but after fluid resuscitation) values. Haemodynamic values at the point of maximum CI response were compared to pre-dobutamine infusion baseline values as correlates of survival to hospital discharge using two-tailed paired Student's t-test analysis. Fisher's Exact test was used for proportional analysis and independent-samples t-tests with Levene correction for unequal group variances was used for continuous measures (Tables 1 and 2). Stepwise discriminant function methods were used to assess the independent impact of 18 haemodynamic and oxygen metabolism variables identified by univariate significance test results (p < 0.2) as potentially associated with survival outcome. Variables defined by discriminant analysis were entered as predictors in a multiple logistic regression model with outcome as the dependent variable (Table 3). Statistical analysis was done with SPSS version 15.0.1. All significance tests are two-tailed. Statistical significance was defined as p < 0.05. Aggregate data is expressed as mean ± standard deviation.

**Table 1:** Baseline Clinical Characteristics and Laboratory Values of Subjects

	All (n = 23)	Non-survivors (n = 15)	Survivors (n = 8)	P value
Male/female	11 M; 12 F	7 M; 8 F	4 M; 4 F	NS
Age	62.6 ± 4.0	61.4 + 5.5	64.8 ± 5.6	NS
Mechanical ventilation	17/23	13/15	4/8	NS
PO_2_/FiO_2_	171.8 ± 20.8	147.2 ± 21.1	217.9 ± 42.4	NS
Pressor use	20/23	13/15	7/8	NS
Dopamine dose (μg/kg/min)	6.8 ± 1.6	7.6 ± 2.5	5.9 + 1.8	NS
Norepinephrine dose (μg/kg/min)	0.22 ± 0.05	0.17 ± 0.05	0.30 ± 0.10	NS
Number of organ failures	5.3 ± 0.3	5.9 ± 0.3	4.3 ± 0.5	0.0138
APACHE II	27.3 ± 1.5	28.9 ± 1.8	24.4 ± 2.6	NS
SAPS	55.2 ± 2.8	61.5 ± 11.3	43.4 ± 2.7	0.0002
Serum creatinine (mg/dL)	2.4 ± 0.3	2.4 ± 0.4	2.4 ± 0.5	NS
Serum albumin (g/dL)	2.3 ± 0.1	2.2 ± 0.1	2.7 ± 0.2	0.0498
Serum bilirubin (mg/dL)	3.2 ± 1.2	4.5 ± 1.7	0.7 ± 0.1	0.0444
Platelets (× 10^3 ^per uL)	171 ± 32	119 ± 31	269 ± 60	0.0483
PT	15.9 ± 0.7	17.1 ± 0.9	13.8 ± 0.7	0.0069
Serum lactate (mmol/L)	2.7 ± 0.3	2.9 ± 0.5	2.5 ± 0.5	NS
Bacteraemia	10/23	7/15	3/8	NS
Organisms	4 S. aureus 3 E.coli 1 S. pneumoniae 1 P. mirabilis 1 C. albicans	4 S. aureus 1 E. coli 1 P. mirabilis 1 C. albicans	2 E. coli 1 S. pneumoniae	
Underlying disorder	5 post-op 4 cancer 2 diabetes 1 each pancreatitis, sickle cell crisis, AIDS, CVA, trauma, cirrhosis, dementia, epilepsy 4 nil	4 post-op 4 cancer 1 each pancreatitis, sickle cell crisis, AIDS, CVA, trauma, cirrhosis, nil	3 nil 2 diabetes 1 each post-op, dementia, epilepsy	
Clinical infection	13 pneu 5 bsi 2 uti 2 peritonitis 1 ssti	7 pneu 5 bsi 1 uti, ssti, peritonitis	6 pneu 1 uti 1 peritonitis	

**Table 2:** Baseline and Dobutamine-Stressed Hemodynamic/Cardiovascular and Metabolic Variables

	Survivor Baseline (n = 8)	Non-survivors Baseline (n = 15)	P value: survivor vs non-survivor-baseline	Survivor: change from baseline	Non-survivor: change from baseline	P value: survivor vs non-survivor-change from baseline
HR (min-1)	88.3 ± 5.1	106.7 ± 5.7	.0254	16.1 ± 4.7	14.9 ± 4.2	.8645
sBP (mmHg)	124.3 ± 6.8	114.0 ± 4.9	.2427	24.6 ± 12.6	8.3 ± 5.2	.1630
dBP (mmHg)	64.1 ± 4.2	57.6 ± 2.8	.2207	-1.6 ± 3.2	2.3 ± 2.4	.3508
PP (mmHg)	60.1 ± 6.5	56.4 ± 4.7	.6541	26.3 ± 10.4	5.9 ± 4.1	.0403
MP (mmHg)	84.2 ± 4.2	76.4 ± 2.8	.1391	7.1 ± 6.0	4.3 ± 3.0	.6225
SVI (ml/beat/m^2^)	31.5 ± 3.0	29.0 ± 3.0	.5663	16.4 ± 3.7	3.0 ± 1.0	.0003
CI (L/min/m^2^)	2.78 ± 0.32	3.01 ± 0.33	.5910	2.24 ± 0.55	0.80 ± 0.22	.0093
RAP (mmHg)	13.6 ± 1.9	14.5 ± 1.4	.7346	-0.8 ± 0.9	0.4 ± 0.8	.9405
sPAP (mmHg)	44.6 ± 4.6	40.5 ± 2.2	.4459	3.5 ± 5.1	2.2 ± 2.1	.6389
dPAP (mmHg)	22.6 ± 2.9	19.5 ± 0.8	.3253	-5.1 ± 1.8	0.6 ± 1.1	.0133
PAM (mmHg)	30.0 ± 3.3	26.5 ± 0.9	.3494	-2.3 ± 2.5	1.1 ± 1.2	.2442
PWP (mmHg)	19.8 ± 2.8	17.6 ± 1.1	.4896	-1.8 ± 1.4	-1.1 ± 0.8	.5829
PAO_2 _(mmHg)	95.6 ± 12.2	97.5 ± 9.4	.9034	-2.3 ± 8.2	-12.2 ± 9.2	.4518
PACO_2 _(mmHg)	35.6 ± 1.5	32.0 ± 2.3	.2000	-1.4 ± 0.9	-0.3 ± 0.8	.3846
O_2_sat (%)	95.2 ± 1.2	96.0 ± 0.7	.4750	-0.6 ± 1.2	-1.0 ± 0.7	.6463
MVO_2 _(%	62.3 ± 3.7	62.1 ± 2.8	.9879	8.8 ± 2.0	2.0 ± 1.6	.0234
LVEF (%)	50 ± 5	57 ± 4	.3184	11.8 ± 3.8	1 ± 2	.0160
LVEDVI (mLm^2^)	65.2 ± 6.5	52.7 ± 5.8	.1691	19.9 ± 7.4	7.8 ± 5.4	.1987
LVESVI (mL/m^2^)	33.6 ± 6.7	23.0 ± 4.4	.2535	3.5 ± 6.5	5.1 ± 5.0	.8497
LVSWI (g.m/m^2^)	28.3 ± 4.1	22.9 ± 2.3	.2567	22.3 ± 7.1	5.2 ± 1.4	.0054
SVRI (dynes/sec/cm^2^/m^2^)	2232 ± 314	1835 ± 170	.2728	-901 ± 246	-162 ± 150	.0154
RVEF (%)	65 ± 3	64 ± 4	.8489	4.8 ± 4.1	2.9 ± 2.5	.6793
RVEDVI (mL/m^2^)	48.4 ± 4.2	46.1 ± 4.7	.7194	25.2 ± 8.8	1.7 ± 2.6	.0047
RVESVI (mL/m^2^)	16.8 ± 2.6	17.4 ± 3.0	.8874	8.7 ± 6.7	-1.0 ± 2.4	.1125
RVSWI (g.m/m^2^)	6.8 ± 1.1	4.5 ± 0.6	.0916	1.8 ± 0.7	1.4 ± 0.6	.4365
PVRI (dynes/sec/cm^2^/m^2^)	322 ± 50	263 ± 45	.3910	-147 ± 39	-21.0 ± 32	.0390
CaO_2 _(mL/dL)	13.4 ± 1.0	12.8 ± 0.5	.6190	-0.1 ± 0.2	-0.1 ± 0.1	.8551
CvO_2 _(mL/dL)	8.6 ± 0.6	8.4 ± 0.6	.8161	1.3 ± 0.4	0.3 ± 0.2	.0208
DO_2_I (mL/min/m^2^)	352 ± 24	383 ± 45	.5448	301 ± 78	98 ± 29	.0084
VO_2_I (mL/min/m^2^)	122 ± 10	122 ± 6	.9967	32 ± 10	22 ± 7	.3652
sbp/lvesvi (mm Hg/mL/m^2^)	4.57 ± 0.73	7.33 ± 1.51	.1152	3.12 ± 1.32	0.15 ± 0.5	.0188
PAS/rvesvi (mm Hg/mL/m^2^)	3.06 ± 0.44	3.21 ± 0.51	.8255	0.69 ± 0.69	0.71 ± 0.90	.9877
lvedvi/pwp (mL/m^2^/mmHg)	3.82 ± 0.67	3.18 ± 0.38	.4123	1.52 ± 0.47	0.74 ± 0.35	.1964
RVEDVI/RAP (mL/m^2^/mmHg)	4.24 ± 0.89	3.44 ± 0.40	.4288	3.63 ± 2.34	0.23 ± 0.46	.0783
pH	7.38 ± 0.3	7.41 ± 0.02	.3988	0 ± 0.01	0 ± 0.01	.8820
Qs/QT	0.11 ± 0.02	0.11 ± 0.02	.8990	0.07 ± 0.05	0.03 ± 0.02	.4793
OER	0.35 ± 0.03	0.35 ± 0.03	.9928	-0.1 ± 0.03	-0.03 ± 0.01	.0160

**Table 3:** Multiple logistic regression results*

	B	SE	Sig	Exp(B)	85% CI	
					Lower	Upper

ΔSVI	0.54	0.27	0.05	1.72	1.32	2.12
ΔsBP/LVESVI	0.45	0.36	0.19	1.57	1.03	2.1
Constant	-5.13	2.24	0.02	0.01		

Overall % Correctly Classified: 95.7% (n = 22/23)

* ΔSVI and ΔsBP/LVESVI identified as unique predictors by discriminant function analysis Structure coefficients: ΔSVI = 0.81, ΔsBP/LVESVI = 0.46

ΔSVI and ΔsBP/LVESVI simultaneous entry with p ≤ 0.15 for inclusion and p ≤ 0.20 for model retention. Overall p = 0.00004, Χ^2 ^= 20.16681, df = 2

Δ = change

## Results

### Subject characteristics

Twenty-three subjects (10 male, 13 female) were recruited for the study. Average (± standard error) age was 62.6 ± 4.0 years. Only eight of twenty-three (35 per cent) patients with severe sepsis survived to hospital discharge. Median and inter-quartile survival duration from the day of the study among non-survivors was six days and two to nine days respectively. No clinically significant adverse effects occurred during the course of the experimental dobutamine challenge. Similar proportions of subjects in each group failed to tolerate the highest planned infusion rate (15 μg/kg/min) of dobutamine (two of eight survivors and five of fourteen non-survivors). In all but one case (a non-survivor), the next highest dose (10 μg/kg/min) was tolerated.

Subject characteristics of the entire group, survivors and non-survivors are shown in Table 1. Survivors and non-survivors were comparable in terms of average age and gender breakdown. Twenty of twenty-three subjects required pressors at the time of entry into the study. Both the proportion requiring pressor support and the degree of pressor requirement was similar in both groups. Inspired oxygen requirement was also similar in both groups. There were no significant differences in the two groups with respect to the arterial partial pressure of oxygen/inspired oxygen fraction (PaO_2_/FiO_2_) ratio. The presence of renal and other individual organ failures was similar between the groups but the mean number of organ failures at the time of study entry was significantly higher in non-survivors. Similarly, the Simplified Acute Physiology Score II (SAPS II) at the time of the study were significantly higher in non-survivors; Acute Physiology and Chronic Health Evaluation II (APACHE II) scores were not. The most common underlying diagnoses in non-survivors were a post-operative state and malignancy. Among survivors, an absence of a major primary underlying disease condition was most common. Pneumonia was the most common underlying infection in both groups accounting for seven of fifteen and six of eight cases in non-survivors and survivors respectively.

### Cardiovascular and haemodynamic variables

**Figure 1:**
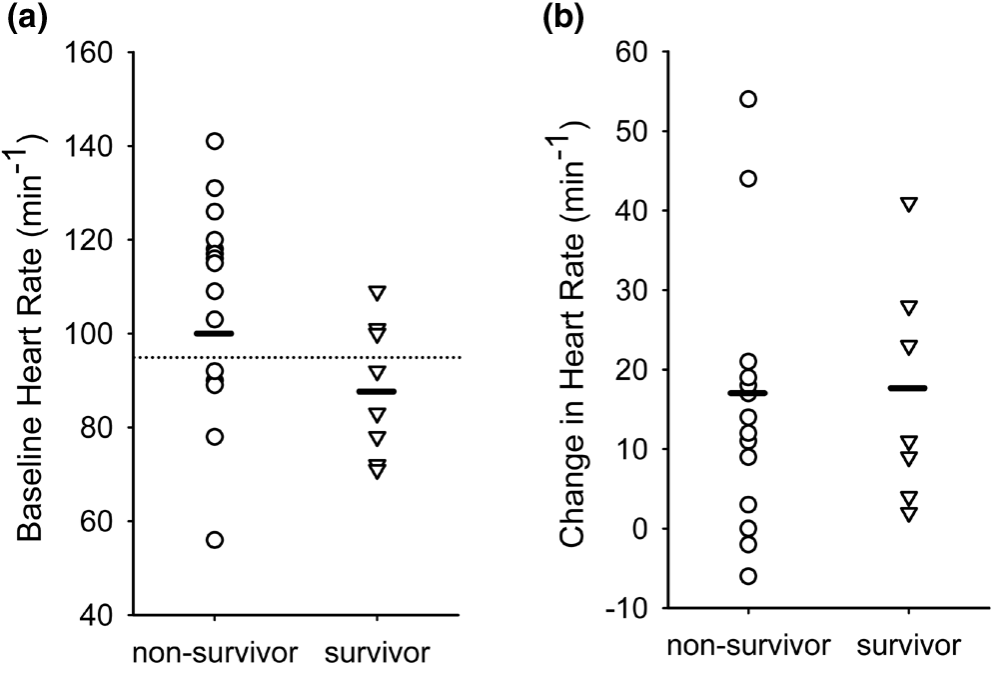
**(a) **Baseline heart rate in survivors and non-survivors. Dotted line indicates optimally predictive cut-off value of 95 beats/min to differentiate between survivors and non-survivors. Outcome stratification was deficient in only 8 of the 23 total subjects using this cut-off. Heart rate was the only baseline haemodynamic variable associated with outcome (p = 0.0254). **(b) **Change in heart rate from baseline at the point of maximal cardiac output response during dobutamine challenge. Change in heart rate was not significantly different between survivors and non-survivors. Dash indicates mean value for group.

Mean difference tests for baseline and dobutamine-stimulated haemodynamic/oxygen transport variables in survivors and non-survivors are shown in Table 2. Among baseline cardiovascular/haemodynamic or oxygen transport variables, only HR was associated with outcome (p = 0.0259). Higher values were associated with increased mortality risk. A cut-off value of 95 beats/min was associated with the optimal correlation with eight mismatches, three survivors and five non-survivors (Table 2, Figure 1a).

**Figure 2:**
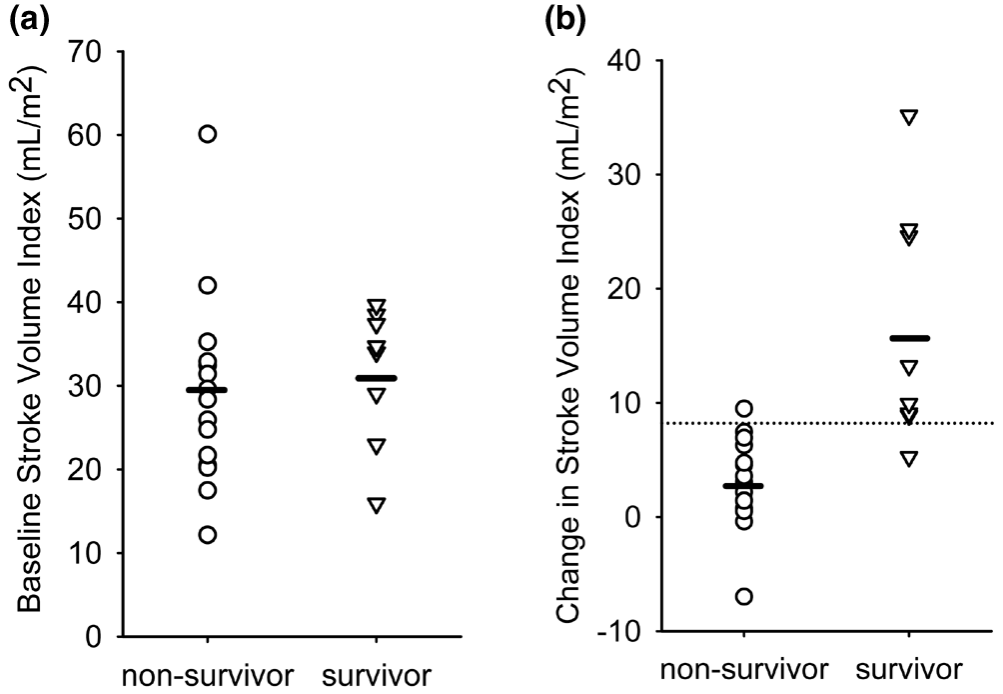
**(a) **Baseline stroke volume index in survivors and non-survivors. Stroke volume index at baseline was not significantly different between survivors and non-survivors. **(b) **Change in stroke volume index from baseline at the point of maximal cardiac output response during dobutamine challenge. Change in stroke volume was the strongest predictor of outcome among tested variables (p = 0.0003). A cut-off value of 8.5 mL/m^2 ^increase in stroke volume index (indicated by the dotted line) correctly categorised outcome in 21 of 23 subjects with only one mismatch in each group. Dash indicates mean values.

**Figure 3:**
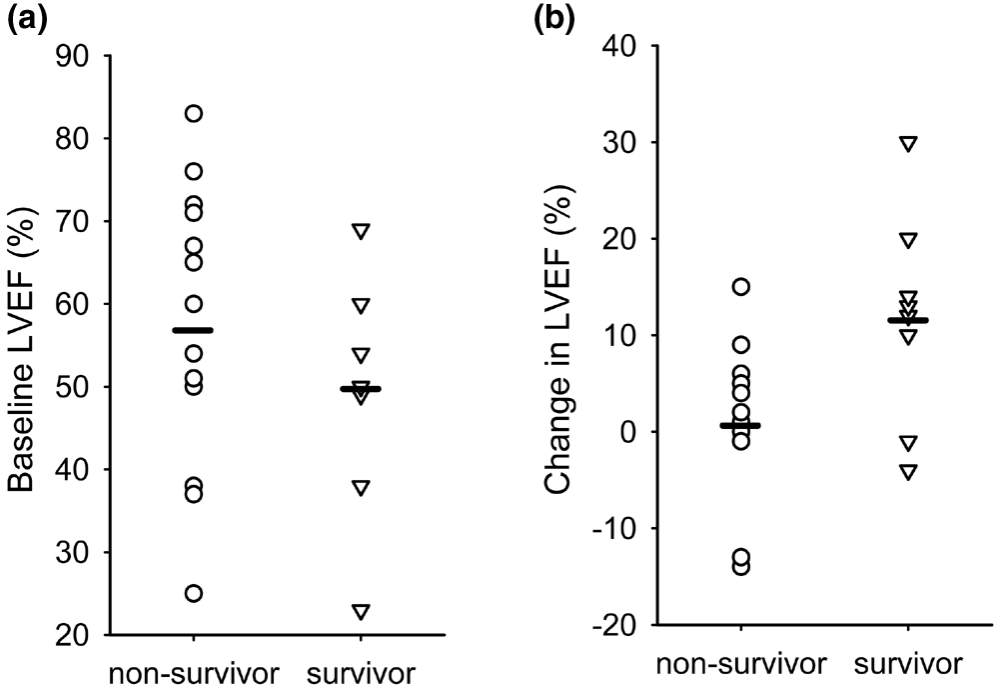
**(a) **Baseline left ventricular ejection fraction in survivors and non-survivors. Seven of fourteen non-survivors and five of eight survivors had a reduced ejection fraction (less than 55 per cent). The lower mean ejection fraction in survivors did not reach statistical significance in comparison to the higher ejection fraction in non-survivors. **(b) **Change in left ventricular ejection fraction from baseline at the point of maximum cardiac index response. The mean increase in left ventricular ejection fraction was significantly greater in survivors than in non-survivors (p = 0.0160). Dash indicates mean values.

**Figure 4:**
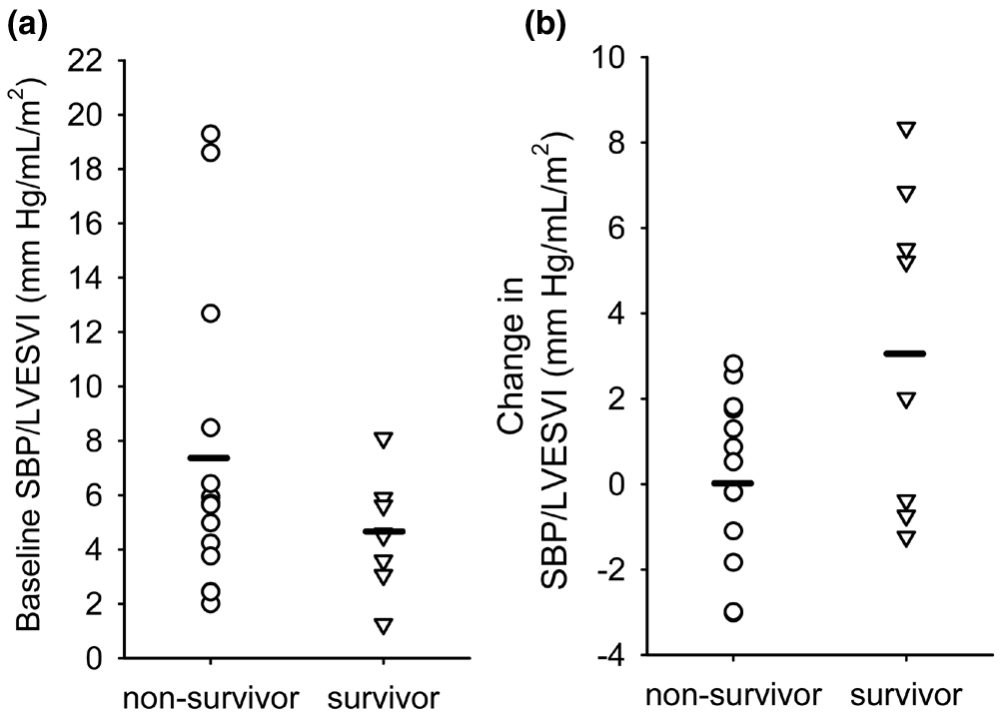
**(a) **Baseline peak systolic blood pressure/left ventricular end-systolic volume index (sBP/LVESVI) in survivors and non-survivors. Baseline sBP/LVESVI was not significantly different between survivors and non-survivors. **(b) **Change in sBP/LVESVI from baseline at the point of maximum cardiac output response. A significantly greater mean increase in sBP/LVESVI was seen in survivors (p = 0.0188). Non-survivors had almost no mean change in this parameter with dobutamine infusion. Dash indicates mean values.

Survivors and non-survivors exhibited divergent responses to dobutamine in several different areas (Table 2, Figure 2a, 3a, 4a, 5a). These areas can broadly be categorised as: 1) cardiac performance including cardiac index (CI) and stroke volume index (SVI); 2) oxygen consumption/delivery including mixed venous oxygen saturation (MVO_2_), venous oxygen content (CvO_2_), oxygen delivery index (DO_2_I) and oxygen extraction ratio (OER); 3) peripheral resistance including diastolic pulmonary artery pressure (dPAP), systemic vascular resistance index (SVRI) and pulmonary vascular resistance index (PVRI); 4) left ventricular contractility including left ventricular ejection fraction (LVEF), peak systolic pressure/left ventricular end-systolic volume index ratio (sBP/LVESVI) and left ventricular stroke work index (LVSWI); and 5) right ventricular lusitropy including right ventricular end-diastolic volume (RVEDVI) and right ventricular end-diastolic volume:right atrial pressure ratio (RVEDVI/RAP).

**Figure 5:**
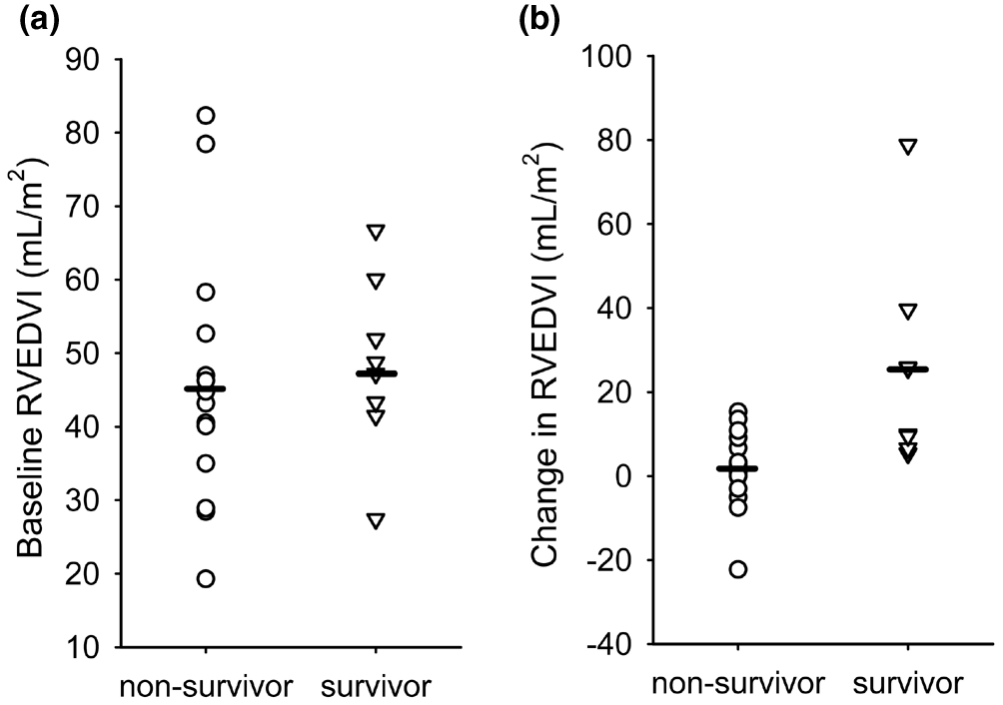
**(a) **Baseline right ventricular end-diastolic volume index (RVEDVI) in survivors and non-survivors. Baseline RVEDVI was similar in both survivors and non-survivors. **(b) **Change in RVEDVI from baseline at the point of maximum cardiac index response. The mean increase in RVEDVI was significantly greater in survivors than non-survivors (p = 0.0047). Dash indicates mean values.

Although baseline heart rate was associated with outcome, heart rate response to dobutamine was not (Table 2, Figure 2b). Both survivors and non-survivors had similar absolute and relative increases in heart rate with dobutamine stimulation. Stroke volume index response had the strongest relationship to outcome with survivors generating more than five-fold more than the approximate mean 3 mL increase generated by non-survivors (Table 2). Interestingly, only two patients demonstrated a decrease in SVI (and CI) with dobutamine infusion. Both were non-survivors. A cut-off value of 8.5 mL/m^2 ^increase in SVI could differentiate between survivors and non-survivors in 21 of 23 cases with just one subject mismatched in each group (Figure 2b). Baseline SVI did not differentiate between survivors and non-survivors (Table 2, Figure 2a).

Other cardiac performance variables related to SVI also were associated with survival on univariate analysis including CI and pulse pressure (PP)(Table 2). Greater increases in DO_2_I, MVO_2 _and CvO_2 _and decreased OER (variables associated with CI) were similarly associated with improved outcome. However, calculated oxygen consumption as reflected by VO_2_I was not. Several variables associated with vascular resistance were also found to be significantly associated with survival on univariate analysis including decreases in SVRI, PVRI and dPAP. Decreases in diastolic systemic arterial pressure did not reach significance in this regard.

In terms of the mechanics of cardiovascular performance, greater increases of left ventricular contractility indices were associated with survival in patients with septic shock (Table 2). Indices of relevance included LVEF (Figure 3), LVSWI and sBP/LVESV (Figure 4). Right ventricular contractility parameters were not similarly associated with survival. However, increases in RVEDVI (Figure 5) and right ventricular compliance (RVEDVI/RAP) either achieved or trended strongly towards significance in association to survival (Table 2).

Using multivariate forward entry discriminant analysis, several alternative models with similar predictive accuracy were identified depending on the significance level used to add and retain variables in each model. In each case, the SVI response was, by far, the most predictive variable. However, the most parsimonious discriminant model achieved maximal accuracy with the addition of sBP/LVESVI response as a 2nd factor in the model. Using this model, outcome in 22 of 23 cases could be appropriately categorised (Table 3). SVI responses were 43 per cent more predictive of outcome than sBP/LVESVI responses in this model (ratio of structure coefficients). Other variables associated with left ventricular contractility and right ventricular lusitropy did not contribute to prediction of outcome in the multivariate model. Multiple logistic model parameters are summarised in Table 3. Positive B coefficients in the logistic regression model indicate that higher SVI and sBP/LVESVI are independently indicative of increased probability of survival. Confidence intervals indicate as much as a doubling of survival odds, on average, over the range of SVI and sBP/LVESVI responses covered by this sample [14]. Discriminant multivariate assessment of relative (indexed to baseline) changes in responses yielded similar results.

## Discussion

Dobutamine, a racemic synthetic catecholamine composed of two enantiomers with divergent sympathetic activity, was developed as an inotropic agent and is most commonly used for support of patients with congestive heart failure and other low output states of cardiogenic decompensation [17]. A series of studies have assessed this compound as a potential adjunctive therapy in severe sepsis for purposes of augmentation of oxygen delivery [18-21]. In addition, a dobutamine stress test with determination of oxygen consumption responses to assess survival potential of patients with severe sepsis and septic shock has been examined [22-24]. No studies have assessed volumetric responses to graded dobutamine infusion in septic patients in relationship to outcome.

This study, like that of Jellema and colleagues [25], shows that dobutamine infusion in patients with septic shock generates a highly heterogenous cardiovascular response. We also demonstrate that dobutamine-stressed ventricular and cardiovascular responses are highly correlated with outcome. The observation that greater increases in left ventricular contractility (LVEF, sBP/LVESVI, LVSWI) and right ventricular lusitropy (RVEDVI, RVEDVI/RAP) indices are associated with survival in severe sepsis and septic shock in univariate analysis, is entirely novel. In addition, an independent contribution to prediction of survival under dobutamine stress from cardiovascular performance (SVI) and left ventricular contractility (sBP/LVESVI) responses has also not been previously reported. Notably, the correlative strength of these responses is substantially more powerful than that generated by initial HR, the only baseline hemodynamic index to predict outcome.

These results are important for at least three reasons. First, they support the notion that simple haemodynamic responses to dobutamine stress can potentially differentiate between survivors and non-survivors of sepsis and septic shock. This confirms long-standing clinical observations and suggests that these responses may be useful in terms of clinical prognostication for purposes of risk stratification in clinical trials. Second, they suggest that dynamic cardiovascular responses may be superior to static performance parameters with respect to their association with survival. Finally, these observations provide important insights regarding the mechanism underlying the cardiovascular response to adrenergic stimulation in survivors versus non-survivors of septic shock.

The observation that only HR among all baselines haemodynamic/oxygen metabolism variables assessed was significantly correlated with outcome has precedence in other studies of septic shock [4,26]. Azimi and coworkers demonstrated that only heart rate among initial haemodynamic variables was associated with outcome with a value of less than 106 beats/min linked to survival [26]. No other initial parameters were associated with prognosis. Parker and colleagues similarly demonstrated that HR of less than 95 beats/min at presentation was associated with survival in a study of 48 patients with septic shock [4]. There are several possibilities to explain the association of an increased HR with a higher probability of death. The increased HR in non-survivors may indicate a higher level of sepsis-induced cardiovascular stress due to a more severe disease state. Alternately, it may represent a secondary compensatory response as a consequence of decreased sensitivity to the direct myocardial inotropic effects of endogenous catecholamines in patient with particularly severe septic myocardial dysfunction [3,27]. Given that baseline SV and sBPVESI was similar in both groups, this possibility seems less likely. Another possibility is that non-survivors have increased sensitivity to catecholamine stimulation. This also seems unlikely since several studies have shown β-adrenergic uncoupling in myocardial tissue exposed to inflammatory mediators such as those found circulating in patients with septic shock [28,29]. Since the study was not designed to determine the cause of this finding, no definitive conclusion can be drawn.

Despite observations dating back several decades suggesting that reduced response to catecholamine support in septic shock is highly predictive of poor outcome, most recent work examining the prognostic significance of dobutamine stress in this situation has focused on oxygen consumption/delivery indices [22-24]. Both Vallet *et al *[24] and Rhodes *et al *[22] have shown that increases of oxygen consumption of greater than 15 per cent with 10 μg/kg/min during severe sepsis are associated with a favorable outcome. However, it is not at all clear that it is necessary to invoke oxygen consumption/delivery responses for predictive power. Both studies clearly demonstrated that cardiac index responses were also highly associated with outcome. However, neither closely examined cardiac performance parameters as correlates of outcome.

In our study, cardiovascular performance as indicated by SVI response was the dominant correlate to outcome with a single cut off value of 8.5 mL/min able to differentiate 21 of 23 outcomes. The predictive power of most other parameters appeared to be physiologically linked to this response and did not persist in multivariate analysis (CI, PP, OER, PAD, SVRI, PVRI, DO_2_I, MvO_2_, CvO_2_). sBP/LVESVI, a relatively load-independent index of left ventricular contractility [30], was the only variable that possessed predictive power independent of stroke volume index (in analysis with both absolute and relative changes from baseline). This response appears to statistically dominate other contractility indices (LVEF and LVSWI) in terms of contribution to the model. If sBP/LVESVI is removed from the model, then LVEF becomes a significant contributor along with SVI.

The fact that both cardiac performance and contractility increases in response to dobutamine stress are highly associated with outcome in septic shock is consistent with the possibility that the presence of *cardiovascular reserve *(that is an indication of submaximal cardiovascular response to baseline stress) may be critical to survival. This concept of preserved *physiologic reserve *as a marker of good prognosis in septic shock has previously been postulated by Hayes *et al *[31] and Rhodes *et al *[22] albeit in the context of oxygen consumption indices. However, it may relate directly to cardiovascular performance and contractility indices as shown in this study.

This study may also demonstrate that the concept of *preload reserve *used most commonly in reference to ischemic heart disease associated limitations in cardiac performance may also apply to septic shock [32]. Several groups of investigators have shown that subjects with clinically asymptomatic impairment of systolic cardiac function can be differentiated from symptomatic patients by the presence of exercise stress-induced increases in SVI in association with ventricular dilatation [33,34]. No direct link has yet been made between survival and the presence of this phenomenon among patients with impaired heart function. Despite this, our study may be unmasking a parallel, related phenomenon in that both exercise and dobutamine challenge represent stressed states in comparison to baseline.

Similarly, our observations of an association of right ventricular dilatation (Table 2, Figure 5a) in response to dobutamine (with a similar trend for the left ventricle) appear to be related to our previous observations which demonstrate a relationship between right and left ventricular dilation and survival in septic shock [3,8,35].

One possibility to tie these observations together is that recruitment of *preload reserve *is a critical but late response during high degrees of cardiovascular stress. If that is the case, then a lack of ability to recruit ventricular volume at any point during cardiovascular stress may indicate that the subject is already operating at the maximum efficacy of the Frank-Starling response and has no additional cardiovascular *reserve*. In this formulation, patients with chronic systolic dysfunction would be more likely to be symptomatic, while patients with septic shock would be more likely to die if such reserve was limited. The nature of the limitation of cardiovascular reserve in conditions of high stress would be a combination of the baseline cardiovascular fitness and the degree of acute cardiovascular stress.

The study demonstrates that cardiovascular hypo-responsiveness to catecholamine stimulation is strongly associated with mortality in severe sepsis and septic shock. Compared to baseline differences in heart rate, dobutamine stress-induced variations in stroke volume responses are much more closely related to outcome. Dobutamine-induced differences in contractility responses also appear to possess independent correlative power with respect to mortality. One physiological mechanism supporting increased cardiac output appears to involve ventricular dilatation in survivors suggesting an important role for "preload reserve" in optimal cardiovascular response to severe sepsis and septic shock. This response may be related to previous observations demonstrating that increased ventricular dilatation is characteristic of survival from septic shock.

This study makes several novel observations. However there are significant limitations to the results. First, despite the fact that dobutamine-stress induced cardiovascular responses are highly associated with outcome, their utility for risk stratification as related to research or clinical prognostication remains unproven. A prospective study would be required to validate their utility for that purpose. Second, these data do not suggest that any of the variables associated with outcome drive these are responsible for these outcomes. It is much more likely that these associations are correlations without causality. For example, reduced stroke volume response to dobutamine likely indicates underlying severity of illness rather than being a direct contributor to risk of death. Finally, there may be significant unmeasured confounders for some of the documented responses. Higher mean intra-thoracic pressures due to more severe lung disease and greater ventilatory requirements could contribute to the smaller RVEDVI and RVEDVI/RAP responses to dobutamine. These patients could also be at higher risk of death. Unfortunately, mean airway pressures are not available for analysis.

## Conclusion

Although a variety of dobutamine-stimulated cardiovascular and ventricular responses are associated with outcome in sepsis and septic shock, SVI increases are most strongly correlated with survival. Since this stimulation test involves transient administration of dobutamine, this response is probably indicative of preservation of cardiac signalling mechanisms and of cardiovascular reserve in the septic patient. Notably, the predictive strength of these responses is substantially more powerful than that generated by initial HR, the only baseline haemodynamic index associated with outcome.

Further research is required to better understand the mechanisms underlying differing cardiovascular responses during severe sepsis and septic shock both at baseline and under exogenous catecholamine stress.

## Key Messages

1) Survival from sepsis and septic shock is closely associated with dobutamine-stimulated increases in stroke volume.

2) Survivors of sepsis and septic shock are characterised by preserved cardiovascular reserve including preload reserve.

## Abbreviations

CI = cardiac index; CO = cardiac output; CvO2 = venous oxygen content; CVP = central venous pressure; DO2I = oxygen delivery index; dPAP = diastolic pulmonary artery pressure; ECG = electrocardiographic; EDV = end-diastolic volume; EF = ejection fraction; ESV = end systolic volume; FiO_2 _= inspired oxygen fraction; HR = heart rate; LVEDVI/PWP = left ventricular end-diastolic volume index: pulmonary wedge pressure ratio; LVEF = left ventricular ejection fraction; LVSWI = left ventricular stroke work index; MAP = mean arterial pressure; mPAP = mean pulmonary artery pressure; MVO2 = mixed venous oxygen saturation; OER = oxygen extraction ratio; PaO_2 _= arterial partial pressure of oxygen; PAP = pulmonary artery pressure; pCO_2 _= partial pressure of carbon dioxide; pO_2 _= partial pressure of oxygen; PP = pulse pressure; PWP = pulmonary wedge pressure; PVR = pulmonary vascular resistance; PVRI = pulmonary vascular resistance index; RAP = right arterial pressure; RVEDV = right ventricular end-diastolic volume; RVEDVI/RAP = right ventricular end-diastolic volume: right atrial pressure ratio; RVEF = right ventricular ejection fraction; RVSWI = right ventricular stroke work index; sBP/LVESVI = peak systolic pressure: left ventricular end-systolic volume index ratio; sPAP/RVESVI = peak systolic pulmonary artery pressure: SV = stroke volume; SVI = stroke volume index; SVR = systemic vascular resistance; SVRI = systemic vascular resistance index; VO_2_I = oxygen consumption index; right ventricular end-systolic volume index ratio.

## Competing interests

The authors declare that they have no competing interests.

## Authors' contributions

Anand Kumar had overall responsibility for this study including study design, administration, and execution. He was also was primarily responsibility for data analysis and manuscript development. Elizabeth Schupp (study execution), Eugene Bunnell (study execution), Amjad Ali (nuclear ventriculography) and Barry Milcarek (statistics) assisted with specific aspects of the project. Joseph Parrillo was involved in study design and manuscript preparation.
